# Potassium *N*-bromo-4-chloro-2-methyl­benzene­sulfonamidate monohydrate

**DOI:** 10.1107/S1600536813014979

**Published:** 2013-06-08

**Authors:** H. S. Spandana, Sabine Foro, B. Thimme Gowda

**Affiliations:** a Department of Chemistry, Mangalore University, Mangalagangotri 574 199, Mangalore, India; b Institute of Materials Science, Darmstadt University of Technology, Petersenstrasse 23, D-64287 Darmstadt, Germany; c Jnanabharathi Campus, Bangalore University, Bangalore 560 056, India

## Abstract

In the title compound, K^+^·C_7_H_6_BrClNO_2_S^−^·H_2_O, the K^+^ cation is hepta­coordinated by two water O atoms, four sulfonyl O atoms of four different *N*-bromo-4-chloro-2-methyl­benzene­sulfonamidate anions, and one Br atom of one of the anions. The S—N distance of 1.584 (3) Å is consistent with an S=N double bond. In the crystal, the anions are linked into layers by O—H⋯Br and O—H⋯N hydrogen bonds.

## Related literature

For preparation of *N*-halo­aryl­sulfonamides, see: Gowda & Mahadevappa (1983 ▶). For studies of the effect of substituents on the structures of *N*-halo­aryl­sulfonamidates, see: George *et al.* (2000 ▶); Gowda *et al.* (2011**a* ▶,b* ▶, 2012 ▶); Olmstead & Power (1986 ▶). For restrained geometry, see: Nardelli (1999 ▶)
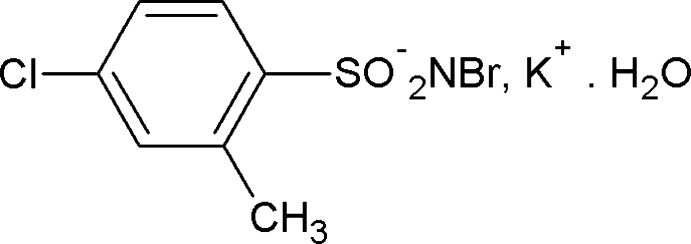


## Experimental

### 

#### Crystal data

K^+^·C_7_H_6_BrClNO_2_S·H_2_O
*M* *_r_* = 340.66
Monoclinic, 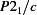

*a* = 15.265 (1) Å
*b* = 11.4817 (8) Å
*c* = 6.7552 (5) Å
β = 101.617 (7)°
*V* = 1159.72 (14) Å^3^
*Z* = 4
Mo *K*α radiation
μ = 4.30 mm^−1^
*T* = 293 K
0.42 × 0.30 × 0.12 mm

#### Data collection

Oxford Diffraction Xcalibur diffractometer with Sapphire CCD detector
Absorption correction: multi-scan (*CrysAlis RED*; Oxford Diffraction, 2009 ▶) *T*_min_ = 0.265, *T*_max_ = 0.627
4387 measured reflections
2367 independent reflections
1971 reflections with *I* > 2σ(*I*)
*R*_int_ = 0.030

#### Refinement

*R*[*F* ^2^ > 2σ(*F* ^2^)] = 0.043
*wR*(*F* ^2^) = 0.129
*S* = 1.10
2367 reflections
143 parameters
3 restraints
H atoms treated by a mixture of independent and constrained refinement
Δρ_max_ = 0.97 e Å^−3^
Δρ_min_ = −1.18 e Å^−3^

### 

Data collection: *CrysAlis CCD* (Oxford Diffraction, 2009 ▶); cell refinement: *CrysAlis CCD*; data reduction: *CrysAlis RED* (Oxford Diffraction, 2009 ▶); program(s) used to solve structure: *SHELXS97* (Sheldrick, 2008 ▶); program(s) used to refine structure: *SHELXL97* (Sheldrick, 2008 ▶); molecular graphics: *PLATON* (Spek, 2009 ▶); software used to prepare material for publication: *SHELXL97*.

## Supplementary Material

Crystal structure: contains datablock(s) I, global. DOI: 10.1107/S1600536813014979/rz5069sup1.cif

Structure factors: contains datablock(s) I. DOI: 10.1107/S1600536813014979/rz5069Isup2.hkl

Supplementary material file. DOI: 10.1107/S1600536813014979/rz5069Isup3.cml

Additional supplementary materials:  crystallographic information; 3D view; checkCIF report

## Figures and Tables

**Table 1:** Hydrogen-bond geometry (Å, °)

*D*—H⋯*A*	*D*—H	H⋯*A*	*D*⋯*A*	*D*—H⋯*A*
O3—H31⋯N1^i^	0.84 (2)	2.00 (2)	2.835 (5)	173 (5)
O3—H32⋯Br1^ii^	0.82 (2)	2.93 (2)	3.744 (3)	173 (4)

Symmetry codes: (i) 
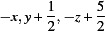
; (ii) 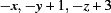.

**Fig. 1.**
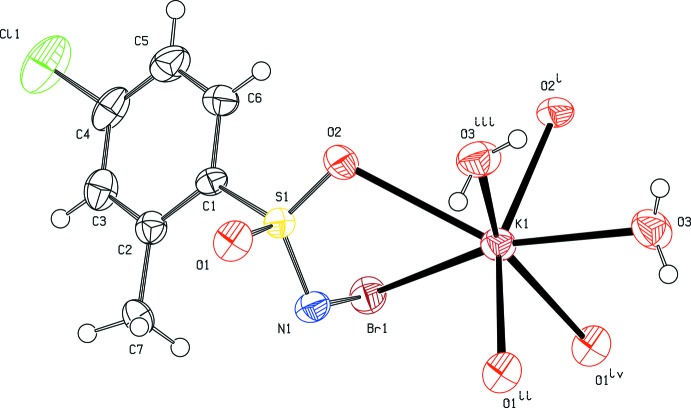
The molecular structure of the title compound, showing the atom labelling extended to show the coordination geometry of the K+ cation. Displacement ellipsoids are drawn at the 50% probability level. H atoms are represented as small spheres of arbitrary radii.

**Fig. 2.**
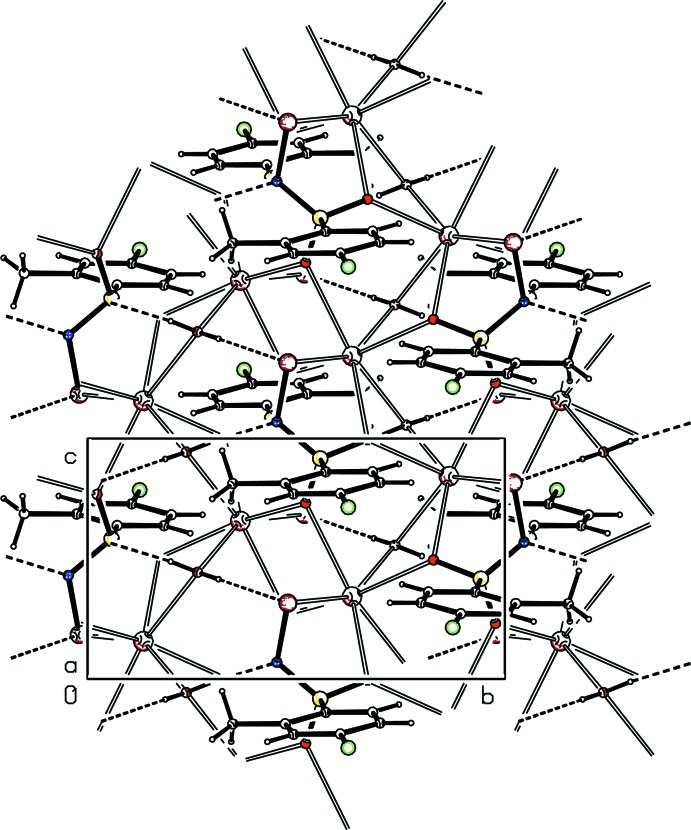
Molecular packing of the title compound with hydrogen bonding shown as dashed lines.

## supplementary crystallographic information

### Comment

The present work was undertaken in order to explore the effect of replacing sodium ion by potassium ion on the solid state structures of metal salts of *N*-haloarylsulfonamidates (Gowda *et al.*, 2011**a*,b*, 2012), the structure of potassium *N*-bromo-2-methyl-4-chlorobenzenesulfonamidate monohydrate (I) has been determined (Fig. 1). The structure of (I) resembles those of potassium *N*-bromo-2,4-dichlorobenzenesulfonamidate sesquihydrate (II) (Gowda *et al.*, 2012), potassium *N*-bromo-4-chlorobenzenesulfonamidate monohydrate (III) (Gowda *et al.*, 2011*a*), potassium *N*-bromo-2-methyl-benzenesulfonamidate sesquihydrate (IV) (Gowda *et al.*, 2011*b*) and other sodium *N*-chloroarylsulfonamidates (George *et al.*, 2000; Olmstead & Power, 1986).

In the title compound, the K^+^ ion is hepta coordinated by two O atoms from two different water molecules, four sulfonyl O atoms of four different *N*-bromo-2-methyl-4-chlorobenzenesulfonamide anions and one Br atom of *N*-bromo-2-methyl-4-chlorobenzenesulfonamide anion, similarly to that observed in III. But this is in contrast to the hepta coordination of K^+^ ion by three O atoms from three different water molecules, four sulfonyl O atoms of three different *N*-bromo-2-methyl-4-chlorobenzenesulfonamide anions in II and IV.

The S—N distance of 1.584 (3) Å is consistent with a S—N double bond and is in agreement with the observed values of 1.575 (3) Å in (II), 1.584 (6) Å in (III) and 1.577 (5) Å in (IV).

In the crystal structure the anions are linked by intermolecular O3—H32···Br1 and O3—H31···N1 hydrogen bonding into layers (Fig. 2 and Table 1).

### Experimental

The title compound was prepared by a method similar to the one described by Gowda & Mahadevappa (Gowda & Mahadevappa, 1983). 2 g of 2-methyl-4-chlorobenzenesulfonamide was dissolved with stirring in 40 ml of 5*M* KOH at room temperature. The resultant solution was cooled in ice and 4 ml of liquid bromine was added drop wise with constant stirring. The resultant potassium salt of *N*-bromo-2-methyl-4-chlorobenzenesulfon-amidate was filtered under suction, washed quickly with a minimum quantity of ice cold water. The purity of the compound was checked by determining its melting point (208° C) and estimating, iodometrically, the amount of active bromine present in it. It was further characterized from its infrared spectrum.

Prism like yellow single crystals of the title compound used in the X-ray diffraction studies were obtained from its aqueous solution at room temperature.

### Refinement

H atoms bonded to C were positioned with idealized geometry using a riding model with the aromatic C—H = 0.93 Å and the methyl C—H = 0.96 Å. The O-bound H atoms were located in difference map and were refined with restrained geometry (Nardelli, 1999), *viz*. O—H distances were restrained to 0.85 (2) Å and H—H distance was restrained to 1.365 Å, thus leading to the angle of 107°. All H atoms were refined with isotropic displacement parameters set at 1.2 *U*_eq_(C-aromatic, O) or 1.5 *U*_eq_(*C*-methyl) of the parent atom. The highest peak and the deepest hole are 0.93 and 0.80 Å from Br1, respectively.

### Crystal data

**Table:**

K^+^·C_7_H_6_BrClNO_2_S·H_2_O	*F*(000) = 672
*M**_r_* = 340.66	*D*_x_ = 1.951 Mg m^−^^3^
Monoclinic, *P*2_1_/*c*	Mo *K*α radiation, λ = 0.71073 Å
Hall symbol: -P 2ybc	Cell parameters from 2484 reflections
*a* = 15.265 (1) Å	θ = 3.1–27.8°
*b* = 11.4817 (8) Å	µ = 4.30 mm^−^^1^
*c* = 6.7552 (5) Å	*T* = 293 K
β = 101.617 (7)°	Prism, yellow
*V* = 1159.72 (14) Å^3^	0.42 × 0.30 × 0.12 mm
*Z* = 4	

### Data collection

**Table:**

Oxford Diffraction Xcalibur diffractometer with Sapphire CCD detector	2367 independent reflections
Radiation source: fine-focus sealed tube	1971 reflections with *I* > 2σ(*I*)
Graphite monochromator	*R*_int_ = 0.030
Rotation method data acquisition using ω scans.	θ_max_ = 26.4°, θ_min_ = 3.3°
Absorption correction: multi-scan (*CrysAlis RED*; Oxford Diffraction, 2009)	*h* = −19→14
*T*_min_ = 0.265, *T*_max_ = 0.627	*k* = −14→14
4387 measured reflections	*l* = −8→8

### Refinement

**Table:**

Refinement on *F*^2^	Primary atom site location: structure-invariant direct methods
Least-squares matrix: full	Secondary atom site location: difference Fourier map
*R*[*F*^2^ > 2σ(*F*^2^)] = 0.043	Hydrogen site location: inferred from neighbouring sites
*wR*(*F*^2^) = 0.129	H atoms treated by a mixture of independent and constrained refinement
*S* = 1.10	*w* = 1/[σ^2^(*F*_o_^2^) + (0.0847*P*)^2^ + 0.3516*P*] where *P* = (*F*_o_^2^ + 2*F*_c_^2^)/3
2367 reflections	(Δ/σ)_max_ = 0.001
143 parameters	Δρ_max_ = 0.97 e Å^−^^3^
3 restraints	Δρ_min_ = −1.18 e Å^−^^3^

### Special details

**Table:**

Experimental. Empirical absorption correction using spherical harmonics, implemented in SCALE3 ABSPACK scaling algorithm.
Geometry. All e.s.d.'s (except the e.s.d. in the dihedral angle between two l.s. planes) are estimated using the full covariance matrix. The cell e.s.d.'s are taken into account individually in the estimation of e.s.d.'s in distances, angles and torsion angles; correlations between e.s.d.'s in cell parameters are only used when they are defined by crystal symmetry. An approximate (isotropic)treatment of cell e.s.d.'s is used for estimating e.s.d.'s involving l.s. planes.
Refinement. Refinement of *F*^2^ against ALL reflections. The weighted *R*-factor *wR* and goodness of fit *S* are based on *F*^2^, conventional *R*-factors *R* are based on *F*, with *F* set to zero for negative *F*^2^. The threshold expression of *F*^2^ > σ(*F*^2^) is used only for calculating *R*-factors(gt) *etc*. and is not relevant to the choice of reflections for refinement. *R*-factors based on *F*^2^ are statistically about twice as large as those based on *F*, and *R*-factors based on ALL data will be even larger.

### Fractional atomic coordinates and isotropic or equivalent isotropic displacement parameters (Å^2^)

**Table:**

	*x*	*y*	*z*	*U*_iso_*/*U*_eq_	
Br1	0.23345 (3)	0.48001 (4)	1.31553 (6)	0.03995 (19)	
K1	0.04696 (6)	0.63387 (8)	1.34287 (13)	0.0345 (2)	
Cl1	0.53340 (9)	0.62486 (17)	0.7141 (2)	0.0736 (5)	
S1	0.15358 (6)	0.55763 (8)	0.91712 (13)	0.0261 (2)	
O1	0.09358 (19)	0.5225 (3)	0.7316 (4)	0.0384 (7)	
O2	0.1350 (2)	0.6710 (2)	0.9950 (4)	0.0373 (7)	
O3	−0.0700 (2)	0.7364 (3)	1.5563 (6)	0.0487 (8)	
H31	−0.091 (3)	0.801 (3)	1.511 (8)	0.058*	
H32	−0.109 (3)	0.694 (3)	1.588 (8)	0.058*	
N1	0.1502 (2)	0.4538 (3)	1.0698 (5)	0.0342 (8)	
C1	0.2632 (2)	0.5708 (3)	0.8616 (5)	0.0252 (7)	
C2	0.3104 (3)	0.4738 (3)	0.8140 (6)	0.0299 (8)	
C3	0.3941 (3)	0.4935 (4)	0.7699 (7)	0.0381 (10)	
H3	0.4277	0.4310	0.7389	0.046*	
C4	0.4281 (3)	0.6058 (5)	0.7717 (6)	0.0412 (11)	
C5	0.3810 (3)	0.7006 (4)	0.8170 (6)	0.0433 (11)	
H5	0.4046	0.7753	0.8170	0.052*	
C6	0.2980 (3)	0.6829 (4)	0.8625 (6)	0.0337 (8)	
H6	0.2651	0.7460	0.8939	0.040*	
C7	0.2770 (3)	0.3482 (3)	0.8127 (6)	0.0330 (9)	
H7A	0.2162	0.3443	0.7399	0.050*	
H7B	0.2801	0.3224	0.9491	0.050*	
H7C	0.3137	0.2989	0.7483	0.050*	

### Atomic displacement parameters (Å^2^)

**Table:**

	*U*^11^	*U*^22^	*U*^33^	*U*^12^	*U*^13^	*U*^23^
Br1	0.0480 (3)	0.0389 (3)	0.0321 (3)	0.00845 (18)	0.00612 (19)	0.00640 (16)
K1	0.0322 (5)	0.0317 (5)	0.0400 (5)	−0.0031 (3)	0.0087 (4)	−0.0016 (4)
Cl1	0.0330 (7)	0.1197 (13)	0.0715 (9)	−0.0165 (7)	0.0187 (6)	0.0105 (8)
S1	0.0229 (5)	0.0261 (5)	0.0291 (5)	0.0015 (4)	0.0047 (3)	0.0014 (4)
O1	0.0251 (15)	0.0519 (18)	0.0348 (16)	−0.0037 (13)	−0.0019 (12)	0.0013 (13)
O2	0.0389 (17)	0.0294 (14)	0.0460 (17)	0.0086 (13)	0.0139 (12)	0.0002 (12)
O3	0.042 (2)	0.0411 (18)	0.066 (2)	0.0001 (15)	0.0176 (15)	−0.0089 (17)
N1	0.0352 (19)	0.0316 (18)	0.0374 (19)	−0.0041 (14)	0.0111 (14)	0.0036 (14)
C1	0.0226 (18)	0.0256 (19)	0.0268 (17)	−0.0038 (15)	0.0039 (14)	0.0022 (14)
C2	0.031 (2)	0.032 (2)	0.0265 (19)	0.0019 (16)	0.0050 (15)	0.0015 (15)
C3	0.029 (2)	0.050 (3)	0.037 (2)	0.0066 (18)	0.0090 (17)	0.0018 (19)
C4	0.022 (2)	0.069 (3)	0.034 (2)	−0.010 (2)	0.0065 (16)	0.007 (2)
C5	0.041 (3)	0.048 (3)	0.041 (2)	−0.018 (2)	0.0081 (18)	0.000 (2)
C6	0.037 (2)	0.029 (2)	0.035 (2)	−0.0032 (17)	0.0048 (16)	0.0009 (16)
C7	0.033 (2)	0.029 (2)	0.038 (2)	0.0080 (16)	0.0091 (16)	−0.0041 (16)

### Geometric parameters (Å, º)

**Table:**

Br1—N1	1.901 (4)	O3—K1^i^	2.786 (4)
Br1—K1	3.3868 (10)	O3—H31	0.842 (19)
K1—O2^i^	2.706 (3)	O3—H32	0.824 (19)
K1—O1^ii^	2.765 (3)	C1—C6	1.392 (5)
K1—O3	2.773 (3)	C1—C2	1.399 (5)
K1—O3^iii^	2.786 (4)	C2—C3	1.387 (6)
K1—O1^iv^	2.878 (3)	C2—C7	1.529 (5)
K1—O2	2.964 (3)	C3—C4	1.389 (7)
K1—N1	3.366 (4)	C3—H3	0.9300
Cl1—C4	1.743 (4)	C4—C5	1.373 (7)
S1—O1	1.453 (3)	C5—C6	1.377 (6)
S1—O2	1.453 (3)	C5—H5	0.9300
S1—N1	1.584 (3)	C6—H6	0.9300
S1—C1	1.794 (4)	C7—H7A	0.9600
O1—K1^ii^	2.765 (3)	C7—H7B	0.9600
O1—K1^v^	2.878 (3)	C7—H7C	0.9600
O2—K1^iii^	2.706 (3)		
			
N1—Br1—K1	73.06 (11)	O3—K1—K1^iii^	104.29 (8)
O2^i^—K1—O1^ii^	156.13 (10)	O3^iii^—K1—K1^iii^	39.16 (7)
O2^i^—K1—O3	76.93 (10)	O1^iv^—K1—K1^iii^	162.22 (6)
O1^ii^—K1—O3	79.22 (10)	O2—K1—K1^iii^	38.44 (5)
O2^i^—K1—O3^iii^	91.20 (10)	N1—K1—K1^iii^	82.90 (6)
O1^ii^—K1—O3^iii^	81.70 (9)	Br1—K1—K1^iii^	98.488 (15)
O3—K1—O3^iii^	75.20 (7)	S1—K1—K1^iii^	57.636 (16)
O2^i^—K1—O1^iv^	90.50 (9)	K1^vi^—K1—K1^iii^	156.16 (3)
O1^ii^—K1—O1^iv^	85.28 (9)	K1^i^—K1—K1^iii^	103.41 (4)
O3—K1—O1^iv^	77.35 (10)	O1—S1—O2	115.20 (18)
O3^iii^—K1—O1^iv^	151.34 (10)	O1—S1—N1	104.79 (19)
O2^i^—K1—O2	84.95 (8)	O2—S1—N1	113.85 (17)
O1^ii^—K1—O2	114.10 (9)	O1—S1—C1	107.20 (17)
O3—K1—O2	142.52 (10)	O2—S1—C1	104.97 (18)
O3^iii^—K1—O2	72.64 (9)	N1—S1—C1	110.72 (18)
O1^iv^—K1—O2	135.98 (9)	O1—S1—K1	116.01 (13)
O2^i^—K1—N1	117.70 (9)	O2—S1—K1	49.88 (12)
O1^ii^—K1—N1	86.17 (9)	N1—S1—K1	65.99 (13)
O3—K1—N1	165.27 (10)	C1—S1—K1	136.12 (12)
O3^iii^—K1—N1	104.65 (10)	S1—O1—K1^ii^	131.95 (17)
O1^iv^—K1—N1	99.80 (9)	S1—O1—K1^v^	131.38 (17)
O2—K1—N1	46.90 (8)	K1^ii^—O1—K1^v^	94.72 (8)
O2^i^—K1—Br1	95.38 (7)	S1—O2—K1^iii^	136.25 (17)
O1^ii^—K1—Br1	106.34 (7)	S1—O2—K1	108.10 (15)
O3—K1—Br1	152.38 (8)	K1^iii^—O2—K1	98.64 (9)
O3^iii^—K1—Br1	132.00 (8)	K1—O3—K1^i^	101.45 (11)
O1^iv^—K1—Br1	76.23 (7)	K1—O3—H31	115 (4)
O2—K1—Br1	60.75 (6)	K1^i^—O3—H31	86 (4)
N1—K1—Br1	32.69 (6)	K1—O3—H32	117 (4)
O2^i^—K1—S1	103.26 (7)	K1^i^—O3—H32	122 (4)
O1^ii^—K1—S1	98.76 (7)	H31—O3—H32	112 (3)
O3—K1—S1	159.84 (8)	S1—N1—Br1	110.23 (19)
O3^iii^—K1—S1	84.65 (8)	S1—N1—K1	88.56 (14)
O1^iv^—K1—S1	122.67 (7)	Br1—N1—K1	74.25 (11)
O2—K1—S1	22.02 (6)	C6—C1—C2	121.6 (3)
N1—K1—S1	25.45 (6)	C6—C1—S1	116.6 (3)
Br1—K1—S1	47.56 (2)	C2—C1—S1	121.8 (3)
O2^i^—K1—K1^vi^	127.95 (7)	C3—C2—C1	117.3 (4)
O1^ii^—K1—K1^vi^	43.70 (6)	C3—C2—C7	118.2 (4)
O3—K1—K1^vi^	73.95 (8)	C1—C2—C7	124.4 (3)
O3^iii^—K1—K1^vi^	120.81 (7)	C2—C3—C4	120.5 (4)
O1^iv^—K1—K1^vi^	41.58 (6)	C2—C3—H3	119.7
O2—K1—K1^vi^	140.40 (7)	C4—C3—H3	119.7
N1—K1—K1^vi^	94.21 (6)	C5—C4—C3	121.8 (4)
Br1—K1—K1^vi^	91.28 (3)	C5—C4—Cl1	119.9 (4)
S1—K1—K1^vi^	118.40 (4)	C3—C4—Cl1	118.4 (4)
O2^i^—K1—K1^i^	42.91 (6)	C4—C5—C6	118.6 (4)
O1^ii^—K1—K1^i^	115.00 (7)	C4—C5—H5	120.7
O3—K1—K1^i^	39.39 (7)	C6—C5—H5	120.7
O3^iii^—K1—K1^i^	96.47 (8)	C5—C6—C1	120.2 (4)
O1^iv^—K1—K1^i^	66.31 (6)	C5—C6—H6	119.9
O2—K1—K1^i^	127.22 (6)	C1—C6—H6	119.9
N1—K1—K1^i^	152.22 (6)	C2—C7—H7A	109.5
Br1—K1—K1^i^	119.924 (16)	C2—C7—H7B	109.5
S1—K1—K1^i^	146.08 (3)	H7A—C7—H7B	109.5
K1^vi^—K1—K1^i^	90.17 (2)	C2—C7—H7C	109.5
O2^i^—K1—K1^iii^	72.93 (7)	H7A—C7—H7C	109.5
O1^ii^—K1—K1^iii^	112.48 (6)	H7B—C7—H7C	109.5
			
N1—Br1—K1—O2^i^	−135.87 (12)	Br1—K1—O2—S1	47.29 (13)
N1—Br1—K1—O1^ii^	54.15 (12)	K1^vi^—K1—O2—S1	−3.2 (2)
N1—Br1—K1—O3	152.19 (19)	K1^i^—K1—O2—S1	154.31 (11)
N1—Br1—K1—O3^iii^	−39.37 (14)	K1^iii^—K1—O2—S1	−144.9 (2)
N1—Br1—K1—O1^iv^	134.94 (12)	O2^i^—K1—O2—K1^iii^	−68.74 (14)
N1—Br1—K1—O2	−54.73 (12)	O1^ii^—K1—O2—K1^iii^	96.32 (10)
N1—Br1—K1—S1	−32.81 (11)	O3—K1—O2—K1^iii^	−8.0 (2)
N1—Br1—K1—K1^vi^	95.82 (11)	O3^iii^—K1—O2—K1^iii^	24.11 (10)
N1—Br1—K1—K1^i^	−173.25 (11)	O1^iv^—K1—O2—K1^iii^	−154.21 (10)
N1—Br1—K1—K1^iii^	−62.37 (10)	N1—K1—O2—K1^iii^	155.05 (16)
O2^i^—K1—S1—O1	−136.58 (16)	Br1—K1—O2—K1^iii^	−167.79 (11)
O1^ii^—K1—S1—O1	34.1 (2)	S1—K1—O2—K1^iii^	144.9 (2)
O3—K1—S1—O1	−48.4 (3)	K1^vi^—K1—O2—K1^iii^	141.75 (6)
O3^iii^—K1—S1—O1	−46.60 (16)	K1^i^—K1—O2—K1^iii^	−60.78 (11)
O1^iv^—K1—S1—O1	124.12 (19)	O2^i^—K1—O3—K1^i^	−25.56 (10)
O2—K1—S1—O1	−102.0 (2)	O1^ii^—K1—O3—K1^i^	155.48 (12)
N1—K1—S1—O1	95.4 (2)	O3^iii^—K1—O3—K1^i^	−120.36 (16)
Br1—K1—S1—O1	138.29 (15)	O1^iv^—K1—O3—K1^i^	67.95 (11)
K1^vi^—K1—S1—O1	75.68 (15)	O2—K1—O3—K1^i^	−88.74 (18)
K1^i^—K1—S1—O1	−140.24 (14)	N1—K1—O3—K1^i^	148.3 (3)
K1^iii^—K1—S1—O1	−77.00 (14)	Br1—K1—O3—K1^i^	50.8 (2)
O2^i^—K1—S1—O2	−34.55 (11)	S1—K1—O3—K1^i^	−118.5 (2)
O1^ii^—K1—S1—O2	136.15 (17)	K1^vi^—K1—O3—K1^i^	110.80 (10)
O3—K1—S1—O2	53.6 (3)	K1^iii^—K1—O3—K1^i^	−93.84 (10)
O3^iii^—K1—S1—O2	55.42 (18)	O1—S1—N1—Br1	175.11 (18)
O1^iv^—K1—S1—O2	−133.86 (18)	O2—S1—N1—Br1	−58.1 (2)
N1—K1—S1—O2	−162.6 (2)	C1—S1—N1—Br1	59.8 (2)
Br1—K1—S1—O2	−119.69 (16)	K1—S1—N1—Br1	−72.62 (15)
K1^vi^—K1—S1—O2	177.70 (17)	O1—S1—N1—K1	−112.27 (15)
K1^i^—K1—S1—O2	−38.22 (17)	O2—S1—N1—K1	14.48 (18)
K1^iii^—K1—S1—O2	25.03 (16)	C1—S1—N1—K1	132.46 (14)
O2^i^—K1—S1—N1	128.05 (16)	K1—Br1—N1—S1	82.43 (18)
O1^ii^—K1—S1—N1	−61.25 (16)	O2^i^—K1—N1—S1	−59.97 (17)
O3—K1—S1—N1	−143.8 (3)	O1^ii^—K1—N1—S1	119.72 (15)
O3^iii^—K1—S1—N1	−141.97 (16)	O3—K1—N1—S1	126.8 (4)
O1^iv^—K1—S1—N1	28.75 (16)	O3^iii^—K1—N1—S1	39.34 (16)
O2—K1—S1—N1	162.6 (2)	O1^iv^—K1—N1—S1	−155.74 (14)
Br1—K1—S1—N1	42.92 (15)	O2—K1—N1—S1	−8.83 (11)
K1^vi^—K1—S1—N1	−19.69 (15)	Br1—K1—N1—S1	−111.50 (18)
K1^i^—K1—S1—N1	124.39 (15)	K1^vi^—K1—N1—S1	162.71 (13)
K1^iii^—K1—S1—N1	−172.37 (15)	K1^i^—K1—N1—S1	−98.89 (17)
O2^i^—K1—S1—C1	32.56 (19)	K1^iii^—K1—N1—S1	6.49 (13)
O1^ii^—K1—S1—C1	−156.73 (19)	O2^i^—K1—N1—Br1	51.53 (13)
O3—K1—S1—C1	120.7 (3)	O1^ii^—K1—N1—Br1	−128.78 (11)
O3^iii^—K1—S1—C1	122.54 (19)	O3—K1—N1—Br1	−121.7 (4)
O1^iv^—K1—S1—C1	−66.74 (19)	O3^iii^—K1—N1—Br1	150.84 (10)
O2—K1—S1—C1	67.1 (2)	O1^iv^—K1—N1—Br1	−44.24 (11)
N1—K1—S1—C1	−95.5 (2)	O2—K1—N1—Br1	102.67 (14)
Br1—K1—S1—C1	−52.57 (18)	S1—K1—N1—Br1	111.50 (18)
K1^vi^—K1—S1—C1	−115.18 (18)	K1^vi^—K1—N1—Br1	−85.79 (9)
K1^i^—K1—S1—C1	28.90 (19)	K1^i^—K1—N1—Br1	12.6 (2)
K1^iii^—K1—S1—C1	92.15 (18)	K1^iii^—K1—N1—Br1	117.99 (9)
O2—S1—O1—K1^ii^	−104.0 (2)	O1—S1—C1—C6	110.7 (3)
N1—S1—O1—K1^ii^	22.0 (3)	O2—S1—C1—C6	−12.3 (3)
C1—S1—O1—K1^ii^	139.7 (2)	N1—S1—C1—C6	−135.6 (3)
K1—S1—O1—K1^ii^	−48.2 (3)	K1—S1—C1—C6	−59.1 (4)
O2—S1—O1—K1^v^	56.0 (3)	O1—S1—C1—C2	−67.5 (3)
N1—S1—O1—K1^v^	−178.1 (2)	O2—S1—C1—C2	169.5 (3)
C1—S1—O1—K1^v^	−60.4 (3)	N1—S1—C1—C2	46.2 (4)
K1—S1—O1—K1^v^	111.75 (18)	K1—S1—C1—C2	122.7 (3)
O1—S1—O2—K1^iii^	−21.0 (3)	C6—C1—C2—C3	0.8 (5)
N1—S1—O2—K1^iii^	−142.1 (2)	S1—C1—C2—C3	178.9 (3)
C1—S1—O2—K1^iii^	96.6 (2)	C6—C1—C2—C7	179.2 (4)
K1—S1—O2—K1^iii^	−124.7 (3)	S1—C1—C2—C7	−2.7 (5)
O1—S1—O2—K1	103.73 (18)	C1—C2—C3—C4	−0.6 (6)
N1—S1—O2—K1	−17.4 (2)	C7—C2—C3—C4	−179.1 (4)
C1—S1—O2—K1	−138.62 (14)	C2—C3—C4—C5	0.1 (7)
O2^i^—K1—O2—S1	146.34 (11)	C2—C3—C4—Cl1	−179.8 (3)
O1^ii^—K1—O2—S1	−48.60 (18)	C3—C4—C5—C6	0.3 (7)
O3—K1—O2—S1	−152.88 (15)	Cl1—C4—C5—C6	−179.8 (3)
O3^iii^—K1—O2—S1	−120.81 (18)	C4—C5—C6—C1	−0.1 (6)
O1^iv^—K1—O2—S1	60.9 (2)	C2—C1—C6—C5	−0.4 (6)
N1—K1—O2—S1	10.13 (13)	S1—C1—C6—C5	−178.6 (3)

Symmetry codes: (i) *x*, −*y*+3/2, *z*+1/2; (ii) −*x*, −*y*+1, −*z*+2; (iii) *x*, −*y*+3/2, *z*−1/2; (iv) *x*, *y*, *z*+1; (v) *x*, *y*, *z*−1; (vi) −*x*, −*y*+1, −*z*+3.

### Hydrogen-bond geometry (Å, º)

**Table:**

*D*—H···*A*	*D*—H	H···*A*	*D*···*A*	*D*—H···*A*
O3—H31···N1^vii^	0.84 (2)	2.00 (2)	2.835 (5)	173 (5)
O3—H32···Br1^vi^	0.82 (2)	2.93 (2)	3.744 (3)	173 (4)

Symmetry codes: (vi) −*x*, −*y*+1, −*z*+3; (vii) −*x*, *y*+1/2, −*z*+5/2.
